# International Expert Consensus on Integrated Skincare Active Ingredients for Pretreatment and Posttreatment Use With Medical Aesthetic Procedures to Enhance Skin Benefits

**DOI:** 10.1111/jocd.70880

**Published:** 2026-05-05

**Authors:** Peter Bjerring, Zoe D. Draelos, Sabrina G. Fabi, Greg J. Goodman, Kwun Cheun Hau, Ariel Haus, Izolda Heydenrych, Jihee Kim, Liang‐Chen Lin, Karim Sayed, Hema Sundaram, Rieko Tsubouchi, Yan Wu, Patricia Brieva, Hina Choudhary, Leihong Flora Xiang

**Affiliations:** ^1^ Department of Dermatology Aalborg University Hospital Aalborg Denmark; ^2^ Dermatology Consulting Services, PLLC High Point North Carolina USA; ^3^ Cosmetic Laser Dermatology, San Diego University of California San Diego Medical Center San Diego California USA; ^4^ Monash University Clayton Victoria Australia; ^5^ Perfect Skin Surgery Centre Hong Kong China; ^6^ Dr Haus Dermatology London UK; ^7^ Cape Town Cosmetic Dermatology Centre, Century City Cape Town South Africa; ^8^ Department of Dermatology, Yongin Severance Hospital Yonsei University College of Medicine Yongin South Korea; ^9^ Bri. Skin Essentials/Brightskin Clinic Hsinchu Taiwan; ^10^ Nomi Oslo Oslo Norway; ^11^ Ouronyx Dubai UAE; ^12^ Private Practice Rockville Maryland USA; ^13^ Private Practice Fairfax Virginia USA; ^14^ Division of Musculoskeletal and Dermatological Sciences, School of Biological Sciences University of Manchester Manchester UK; ^15^ Ginza Skin Clinic Tokyo Japan; ^16^ Department of Dermatology Peking University First Hospital Beijing China; ^17^ SkinCeuticals New York New York USA; ^18^ Huashan Hospital Fudan University Shanghai China

**Keywords:** consensus, dermocosmetics, integrated skincare, periprocedural, postprocedural, skin barrier repair, skincare ingredients

## Abstract

**Background:**

Integrated skincare (ISC) combines dermocosmetics with medical aesthetic procedures to improve outcomes. However, guidance on selecting appropriate active ingredients across different procedure types and phases remains limited.

**Aims:**

To provide international guidance on appropriate ISC actives for use with aesthetic procedures.

**Methods:**

In a simplified Delphi study, 14 experts evaluated 44 actives for use across four procedure categories (ablative energy‐based, non‐ablative energy‐based, non‐energy‐based procedures with or without skin barrier disruption) at four time points (pretreatment, peri‐treatment, aftercare, and follow‐up ≥ 1 week post‐procedure). The panelists also ranked their top five preferred actives for each procedure category and time point.

**Results:**

Ceramides, cholesterol, hyaluronic acid, niacinamide, and peptides were deemed appropriate across all procedure categories and all time points. Azelaic acid, benzoyl peroxide, cysteamine, glycolic acid, hydroquinone, lactic acid, retinoids, and salicylic acid were identified as unsuitable for use on treatment day. Ceramides and hyaluronic acid were highly ranked for pre‐treatment, peri‐treatment and aftercare, while retinoids and ferulic acid were highly ranked for pretreatment and followup. Vitamin C was ranked in the top 5 across all procedure categories and time points, except at pretreatment for non‐energy‐based without skin barrier damage.

**Conclusions:**

Safety and tolerability are primary considerations when selecting ISC actives. Ingredients with higher irritation potential, such as retinoids and acids, should be avoided during the healing phase (on treatment day and during short‐term aftercare) due to risk of irritation, as well as post‐inflammatory hyperpigmentation in skin of color.

## 1 Introduction

Integrated skincare (ISC) combines the use of clinically proven skincare or dermocosmetic products as adjuncts before, during, and after medical aesthetic procedures to prepare, protect and repair the skin, thereby supporting improved long‐term aesthetic outcomes. ISC for skin rejuvenation is especially beneficial for patients with concomitant skin conditions such as acne or acne scarring, rosacea, melasma and hyperpigmentation, or dyschromia [1].

Aesthetic procedures, even when non‐invasive with short downtime and reduced risks, may cause skin barrier disruption, inducing pathological and physiological processes such as oxidative stress and inflammation. Although most adverse effects are transient, potential complications include post‐inflammatory hyperpigmentation (PIH) or hypopigmentation, worsening of melasma or acne, microbial infection, erythema, skin pain, and scarring. The risk of PIH or hypopigmentation is greatest in skin of color (SOC) and acne‐prone skin or other conditions characterized by chronic inflammation, especially following more invasive treatments such as ablative laser treatments, radiofrequency microneedling (RFMN) or chemical peels [2, 3]. In addition to using pre‐procedure sun‐protection, incorporating periprocedural ISC, such as antioxidants, anti‐inflammatory agents, and ingredients that promote skin barrier repair, may help mitigate or prevent potential side effects while enhancing outcomes [4, 5].

Combining high‐quality topical ingredients with aesthetic procedures is aimed at minimizing discomfort, promoting healing, and reducing downtime, while improving cosmesis and patient satisfaction. However, given the ever‐increasing number of actives in skincare formulations, the wide range aesthetic procedures performed for various cosmetic or therapeutic purposes (e.g., skin rejuvenation, acne scars, pigmentation disorders, rosacea), and the increased emphasis on ISC in dermatology, there is a need for evidence‐based guidance to identify appropriate actives for ISC.

A simplified Delphi method inspired by the RAND/UCLA Appropriateness Method (RAM) [6] is a useful expert‐guided approach when there is a paucity of clinical evidence from randomized controlled trials (RCT).

The primary objective of this international expert consensus was to provide standardized and updated guidance on the appropriateness of a comprehensive range of topical ISC actives used as adjuncts for a broad range of aesthetic procedures, including ablative energy‐based (e.g., CO_2_ or fractional CO_2_ laser, Er:YAG), non‐ablative energy‐based (e.g., nano or picosecond Nd:YAG, intense pulsed light [IPL], dye lasers, ultrasound, radiofrequency microneedling [RFMN]), non‐energy‐based without barrier disruption (e.g., topical retinoids, topical/injected hyaluronic acid, neuromodulator injections, injectable fillers) and non‐energy‐based with barrier disruption (e.g., microneedling, chemical peels).

## 2 Methods

### 2.1 Literature Search Strategy

Actives and situations were identified by a focused PubMed literature search conducted in 2025 with no date restrictions. Eligible publications included English language clinical studies on humans and consensus or guideline studies addressing the use of ISC ingredients in conjunction with aesthetic procedures.

### 2.2 Consensus Studies

Search terms for consensus study publications included: consensus, algorithm, integrated skincare, cosmetic, dermatology, dermocosmetic, energy‐based, non‐energy, periprocedural, experts, expert opinion, panel, Delphi.

We identified 32 relevant consensus guidelines or algorithm publications covering ISC used as adjuncts for 10 different aesthetic procedures or treatments and 8 dermatologic indications (skin rejuvenation, scars, pigmentation disorders, vitiligo, acne, rosacea, melasma, and atopic dermatitis).

### 2.3 Integrated Skincare Studies

To conduct an extensive review of the literature, search terms used for the various scenarios included: (procedure) OR (procedure synonym) AND (connector) AND (cosmetic/study term). For example, (Er:YAG) OR (erbium‐doped yttrium aluminum garnet) AND (topical) AND (split‐face). We limited most procedural categories to 5 studies.

Key terms for procedures included: Er:YAG laser, Nd:YAG laser, picosecond laser, non‐ablative laser, fractional ablative laser (or CO_2_ laser), RFMN, ultrasound, retinoids, hyaluronic acid, neuromodulator injections (or neuromodulator filler combined approach), injectable fillers (or calcium hydroxylapatite/polymethylmethaacrylate to exclude HA), microneedling, facial peel. Connector terms included: in combination with, laser‐assisted, assisted delivery. Cosmetic study terms included: topical, serum, cream, antioxidant, split‐face, cosmetic, skincare, dermocosmetic, facial. Additional active‐specific terms included: vitamin C, vitamin E, and acids (hyaluronic, tranexamic, salicylic).

Preference was given to facial studies with high‐quality design (e.g., RCT over retrospective; comparative split‐face over non‐comparative observational studies), particularly more recent and/or highly cited studies.

We retrieved 68 ISC clinical studies (46 RCT), published between 2003 and 2025. Of these, 49% enrolled both men and women, while 66% included patients with SOC. Based on the literature search, common clinical practice, and panelist knowledge of emerging ingredients, a list of 44 actives was compiled for the survey (Tables 1 and S1).

**TABLE 1:** Integrated skincare actives and procedures based on the literature search and expert clinical practice.

Group	Procedure	Mechanism/Barrier disruption	Common indications	Primary concerns	Cosmetic actives
Energy‐based Ablative Lasers	Fractional Ablative Er:YAG	Mild photothermal heating of dermis; low epidermal disruption	Mild wrinkles, superficial scars	Mild erythema, transient sensitivity	Growth factors/Vitamin C [7]
					Hydroquinone [8, 9, 10]
					Tranexamic acid (intradermal) [10]
	CO_2_ Laser	High thermal energy vaporizes skin layers; major disruption	Deep wrinkles, scars, photoaging	Erythema, PIH, infection, downtime	Poly‐L‐lactic acid (PLLA) [11]
					Tranexamic acid [12]
					Vitamin C/Vitamin E/Ferulic acid [13]
					Vitamin C [14]
Energy‐based Non‐Ablative	Radiofrequency with or without Microneedling	Microneedles create micro‐injuries; RF energy heats dermis; temporary barrier disruption enhances absorption	Acne scars, fine lines, skin laxity, uneven texture, hyperpigmentation	Redness, swelling, dryness, PIH	Botanical Extracts [15]
					Ceramides/Cholesterol/Fatty acids [16]
					Hyaluronic acid [17]
					Lactic hydroxyacid/Yeast extract/Tripeptide/Hydrolyzed rice protein [18]
					Peptides [19]
					PLLA [20]
	Nd:YAG	Deep dermal heating; collagen remodeling; minimal epidermal effect	Pigmentation, vascular lesions, tightening	Hyperpigmentation, mild edema	Alpha arbutin [21]
					Azelaic acid [22]
					Hydroquinone [23, 24, 25]
					Tranexamic acid/Kojic acid/Niacinamide/HEPES [26]
					Tranexamic acid [27]
					Vitamin C/Vitamin E/Ferulic acid [28]
					Vitamin C [29, 30]
	Intense Pulsed Light/1927‐nm Diode Laser/Thulium 1927‐nm Fractional Laser/Non‐ablative Fractional Resurfacing	Heats dermal tissue without epidermal removal; preserves skin barrier	Melasma, photodamage, moderate wrinkles, telangiectasia	Erythema, hyperpigmentation	Botanical extracts (Thyme/Olive/Cucumber) [31]
					Hydroquinone [32]
					Panthenol/Bisabolol [33]
					Peptides [34]
					Resveratrol/Baicalin/Vitamin E [35]
					Resveratrol [31]
					Silymarin/Salicylic acid/Vitamin C/Ferulic acid [36]
					Tranexamic acid [37, 38]
					Vitamin C/Vitamin E/Ferulic acid [35, 39]
	Picosecond	Photoacoustic effect; mechanical disruption; minimal thermal damage; barrier mostly spared	Pigmented lesions, melasma, acne scars, fine lines	Redness, swelling, peeling	Hydroquinone [40, 41, 42]
					Tranexamic acid [43, 44]
	High Frequency Ultrasound	Thermal stimulation via ultrasound; no ablation; intact barrier	Laxity, fine lines, dermal remodeling, acne scars, melasma	Transient redness, rare swelling	Bisabolol/Vitamins C and E/Peptides [45]
					Glutathione/Hyaluronic acid [46]
					Peptides/Ferulic acid/Resveratrol/Botanical extracts/Fatty acids [47]
					Vitamin C/Ferulic acid/Phloretin [48]
					Niacinamide/Vitamin C [49]
					Wild fruit flavonoids/Proxylane (C‐xyloside)/Rhamnose [50]
Non‐Energy‐Based Without Barrier Disruption	Injectables (e.g., neuromodulators, fillers)	Intradermal delivery or neuromodulation; no epidermal disruption	Wrinkles, volume loss, muscle relaxation, scars	Bruising, swelling, lumpiness	Adenosine/Retinol/Hyaluronic acid [51]
					Exosomes [52]
					Hydroquinone/Retinoids [53]
					Peptides/Niacinamide/Polyhydroxy acid/Laminaria extract [54]
					Vitamin C [55]
	Topical Retinoids	Cellular turnover via nuclear receptor activation; no barrier injury	Acne, aging, hyperpigmentation	Irritation, purging, photosensitivity	Benzoyl peroxide [56, 57]
					Glycolic acid [58]
					Silymarin/Salicylic acid/Vitamin C/Ferulic acid [59]
Non‐Energy‐Based With Barrier Disruption	Microneedling (physical)	Microchannels created in skin; mechanical barrier disruption	Scars, texture, aging, pigmentation, melasma	Infection risk, PIH, irritation	Hydroquinone [60] Tranexamic acid [60, 61]
					Vitamin C [29, 62]
	Chemical Peels (e.g., glycolic acid)	Controlled chemical exfoliation of epidermis; moderate disruption	Melasma, acne, fine lines, dyschromia	Peeling, burning, PIH	Azelaic acid [63]
					Salicylic acid/Retinoic acid [64]
					Tranexamic acid [65]
					Vitamin C [66, 67]

### 2.4 International Expert Panel

The international panel comprised 14 experts with published credentials (13 board‐certified dermatologists and 1 aesthetic doctor) from 10 countries across 5 continents. All have extensive experience (over 10 years) of integrating skincare with dermatologic and aesthetic procedures and the use of periprocedural ISC to prevent complications such as PIH. A live meeting was convened on 28 March 2025 (10 in‐person in Monte Carlo, Monaco and 4 virtual attendance) to discuss the existing evidence on ISC actives. Groups of 5 panelists were each assigned one procedure category (non‐ablative energy‐based, ablative energy‐based, or non‐energy‐based procedures with or without skin barrier disruption) and indicated whether they would recommend the active at the respective time point by voting “yes” or “no”.

### 2.5 Survey

During April–May 2025, all panelists completed an online survey rating the 44 ISC active ingredients on a 4‐point scale (1 = Never appropriate, 2 = Rarely, 3 = Sometimes, 4 = Often/Always) for all four procedure categories across four treatment periods. Responses were dichotomized into appropriate (“Often/Always” or “Sometimes appropriate”) or not appropriate (“Never” or “Rarely appropriate”) and analyzed using a simplified Delphi method, inspired by the RAND/UCLA method adapted to a 4‐point evaluation [6].

Consensus was defined as follows: appropriate if ≥ 11 panelists (if fewer than 14 panelists responded, consensus was defined as ≥ 75% agreement among respondents) voted “Often/Always” or “Sometimes appropriate” (with < 4/14 answers in “Never” or “Rarely appropriate”); not appropriate if ≥ 11 panelists (or ≥ 75% agreement among panelists who responded) voted “Never” or “Rarely appropriate” (with < 4/14 answers in “Often/Always” or “Sometimes appropriate”). All other scenarios were classified as no consensus reached. Of note, the lowest consensus score of 11 votes versus 3 votes met statistical significance (*p* = 0.03; one‐sample chi‐square test).

The survey also asked panelists to list their top 5 recommended actives for each procedure category and each time point. Ranking of actives was performed as follows: (i) number of times ranked in the top 5; (ii) number of times ranked in the top 3; and (iii) overall mean ranking score (from 0 = active not selected to 5 = active ranked in first position). Following collegiate decision, the mean score (higher score = higher rank) served as the primary basis for ranking, with ties resolved by the total number of top‐5 votes across all panelists; any remaining ties were assigned equal rank.

Panelists could also provide free‐text comments on the actives.

A second virtual meeting was held in June 2025 to discuss, refine, and validate the consensus results.

## 3 Results

### 3.1 Consensus Results

**FIGURE 1:**
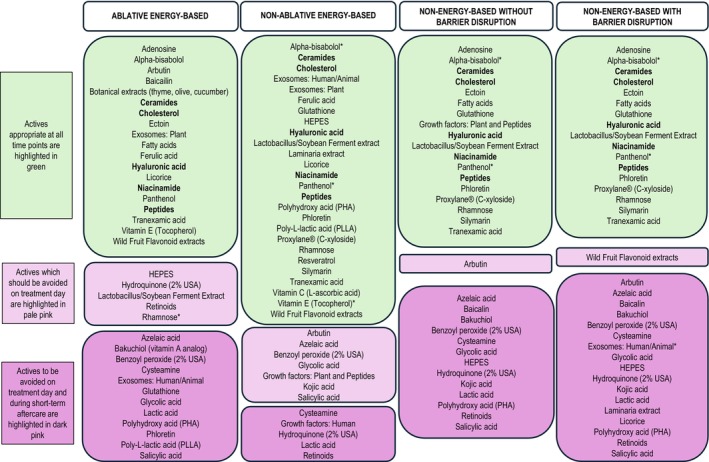
Consensus results for ISC actives. Actives in bold text have consensus to use at all time points across all four procedure categories. Data was collected for 44 actives across 4 time points (pretreatment [Day −14 to −1], treatment day [Day 0], aftercare [Day 1–7] and followup [>Day 7–28]) and 4 procedure categories. For each specific procedure category, actives appropriate at all time points are highlighted in green, actives which should be avoided on treatment day are highlighted in pale pink, and actives to be avoided on both treatment day and during short‐term aftercare are highlighted in dark pink. Examples of the different procedure categories include ablative energy‐based (e.g., fractional CO_2_ laser, Er:YAG), non‐ablative energy‐based (e.g., nano or picosecond Nd:YAG, intense pulsed light, radiofrequency microneedling), non‐energy‐based non‐disruptive (e.g., fillers, neuromodulators) and non‐energy‐based barrier disruptive (e.g., microneedling, chemical peels). *Active ingredients where at least one time point had fewer than 14 panelist responses; consensus was defined as ≥ 75% agreement among respondents. Abbreviations: HEPES Hydroxyethylpiperazine Ethane Sulfonic Acid, PHA Polyhydroxy acid, PLLA Poly‐L‐lactic acid.

Consensus results for each procedure category are shown in Figure 1. Several actives were deemed appropriate across all time points, whereas others were identified as unsuitable for use on treatment day and during short‐term aftercare (Figure 1). Ceramides, cholesterol, hyaluronic acid, niacinamide, and peptides were deemed appropriate across all four procedure categories and all time points (shown in bold in Figure 1). Despite incomplete responses for actives alpha‐bisabolol and panthenol at some time points, both were considered appropriate across all procedure categories and all time points, based on a consensus of ≥ 75% agreement among respondents. The number of respondents for active ingredients that reached consensus but had at least one time point with fewer than 14 panelist responses is provided in Table S2. Certain actives, including kojic acid, laminaria extract, resveratrol, and vitamin C, were recommended for situational use for ablative energy‐based procedures at all time points except for short‐term aftercare.

Azelaic acid, benzoyl peroxide, cysteamine, glycolic acid, hydroquinone, lactic acid, retinoids, and salicylic acid were identified as unsuitable across all four procedure categories for use on treatment day (and, in some cases, also during short‐term aftercare). Benzoyl peroxide (2% USA) was inappropriate at all time points for ablative energy‐based and both non‐energy‐based categories (with and without barrier disruption).

Actives that did not reach consensus at one or more time points within certain procedure categories, including some growth factors, exosomes, and plant extracts, are detailed in Table S3.

### 3.2 Top Ranked Recommended Actives

**FIGURE 2:**
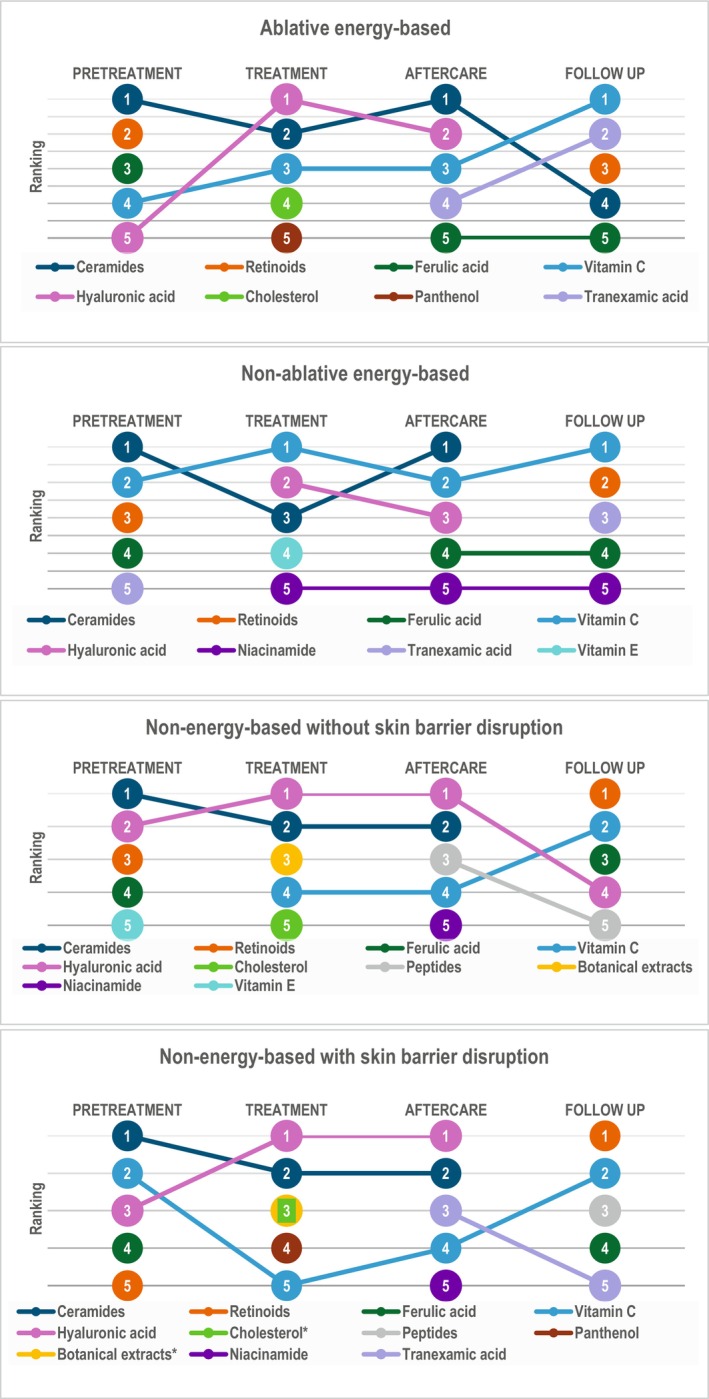
Top five recommended actives at each time point for ablative energy‐based procedures, non‐ablative energy‐based procedures, non‐energy‐based procedures without skin barrier disruption, and non‐energy‐based procedures with skin barrier disruption. Experts selected their top 5 actives for each procedure category and each time point as follows: (i) number of times ranked in the top 5; (ii) number of times ranked in the top 3; and (iii) overall mean ranking score (from 0 = active not selected to 5 = active ranked in first position). The mean score (higher score = higher rank) served as the primary basis for ranking, with ties resolved by total number of top‐5 votes across all panelists; any remaining ties were assigned equal rank. *Equal‐ranking positions.

Based on expert opinion, the top 5 recommended ISC actives for each procedure category and each time point are summarized in Figure 2. Certain actives were ranked consistently in the top 5 across all 4 procedure categories: ceramides, ferulic acid, and retinoids at pretreatment; ceramides, hyaluronic acid, and vitamin C on treatment day and short‐term aftercare; ferulic acid, retinoids, and vitamin C at followup.

In summary, ceramides and hyaluronic acid were highly ranked throughout the treatment and short‐term aftercare phases, whereas retinoids and ferulic acid were highly ranked at pretreatment and followup. Vitamin C was ranked in the top 5 across all procedure categories and time points, except at pretreatment for non‐energy‐based without skin barrier damage.

In addition to ceramides, hyaluronic acid, and vitamin C, other actives ranked in the top 5 at treatment day included cholesterol (for all procedure categories except non‐ablative energy‐based), panthenol (ablative energy‐based, non‐energy‐based with skin barrier damage), vitamin E, niacinamide (non‐ablative energy‐based), and botanical extracts (non‐energy‐based with or without skin barrier damage).

## 4 Discussion

Most existing clinical studies evaluated a limited number of actives (or formulations) with one specific aesthetic procedure (see Table 1). To extend the guidance across several types of aesthetic procedures, this international consensus assessed 44 ISC actives for energy‐based (ablative and non‐ablative) and non‐energy‐based aesthetic procedures (with or without skin barrier disruption) at four time points (before treatment, treatment day, aftercare, and followup from 1‐week post‐procedure).

Actives with a positive consensus result across all four procedure categories and all time points included ceramides, cholesterol, hyaluronic acid, niacinamide, panthenol, and peptides. Of note, variations in quality and molecular weight of hyaluronic acid will influence safety and tolerability since low molecular weight hyaluronic acid may penetrate deeper to promote healing and skin barrier repair but may also be more inflammatory, particularly in the context of ablative procedures.

To combine quantitative analysis with qualitative insights, the experts selected their top 5 recommended actives for each situation. Ceramides, hyaluronic acid, and vitamin C were highly ranked for all four procedure categories at treatment and aftercare for their anti‐inflammatory and soothing properties, as well as a good tolerability profile. Ferulic acid was ranked in the top 5 at both pretreatment and followup for all procedure categories, as well as during aftercare for energy‐based procedures. Ceramides and antioxidants (vitamin C, ferulic acid) may improve skin quality and enhance outcomes. Other actives ranked in the top 5 for use on treatment day with certain procedures included cholesterol, panthenol, vitamin E, niacinamide, and botanical extracts, which may be well‐tolerated depending on the procedure and the degree of skin barrier disruption. Indeed, some actives may penetrate deeper after mild to moderate barrier disruption and become more potent, while also helping to support barrier repair [4].

As safety is the primary concern during the healing phase until complete re‐epithelialization and skin barrier repair, the panelists recommended avoiding some actives (e.g., retinoids, azelaic acid, glycolic acid, lactic acid, salicylic acid, benzoyl peroxide [2% USA], cysteamine, or hydroquinone [2% USA]) immediately after the procedure due to a risk of inflammation and irritation, contact dermatitis, or hyperpigmentation in SOC. Despite a strong consensus to avoid retinoids on treatment day and during short‐term aftercare due to irritation concerns, retinoids were ranked in the top 5 recommended actives at pre‐procedure and during long term followup for all procedure categories. Panelists discussed the superiority of retinoic acid over other retinoids for increasing cell turnover and collagen production to promote skin renewal and rejuvenation. Regulatory restrictions in some countries may explain the recommendation to avoid hydroquinone on treatment day, especially as effective alternatives exist for preventing pigmentation (e.g., tranexamic acid, kojic acid, vitamin C, niacinamide) [2, 68, 69]. For example, a high incidence of rebound PIH was observed when treating telangiectatic melasma with a combination of non‐ablative energy‐based laser and 4% hydroquinone cream in patients with SOC [70].

Consensus was not reached for some actives for certain procedure categories and time points, for example some plant extracts, exosomes, and growth factors (Table S3). Essential oils and botanical extracts (or combinations) are two families of ingredients derived from plants by extraction or distillation; essential oils are lipophilic, whereas botanical extracts can also be aqueous and alcoholic. Variability in source, purity, or extraction may increase the risk of contact dermatitis, highlighting the importance of obtaining clinical evidence to confirm the tolerability and effectiveness for specific formulations. For example, thyme has antimicrobial and anti‐inflammatory properties, but only preparations with a low thymol content should be used during or immediately after procedures involving ablation or barrier disruption. Similarly, cucumber extracts obtained via alcohol‐based methods may be irritating.

Clinical evidence suggests that specific botanical formulations may provide cooling, soothing, and healing effects on irritated skin to accelerate skin barrier repair when used as ISC with energy‐based or RFMN aesthetic treatments [4, 15]. Notably, in an open‐label, split‐face trial, a mask formulation enriched with cucumber and thyme extracts (Phyto Corrective Masque, SkinCeuticals, NY, USA), used in conjunction with a resveratrol‐based antioxidant serum (Resveratrol B E, SkinCeuticals), demonstrated significant anti‐inflammatory, antimicrobial, and antioxidant properties. This regimen was shown to mitigate post‐procedure erythema and improve patient comfort during intense pulsed light treatment for rosacea [31].

Exosomes, which are extracellular nano‐sized vesicles, serve as delivery systems for actives for promoting skin rejuvenation, for example. A comparative study in 40 patients found that when the epidermis and dermis were primed with topical placental mesenchymal stem cell‐derived exosomes (mixed with hyaluronic acid filler and botulinum toxin) prior to calcium hydroxylapatite injections (CaHA), skin quality improvements were enhanced [52]. A small case series of 3 patients suggested that topical human mesenchymal stem cell‐derived exosomes may accelerate post‐procedural skin barrier restoration and wound healing [71]. However, the panelists stressed the importance of product quality and bioavailability and the need for further clinical evidence before recommending them as ISC with aesthetic procedures.

Similarly, while some RCT evidence suggests that topical human growth factors may be appropriate as adjuncts to skin rejuvenation procedures during the treatment and aftercare phases [7, 72], product quality for specific growth factors is crucial.

### 4.1 Limitations

The main limitations of this study include the absence of a systematic review, the relatively small number of panelists, and the use of a simplified Delphi approach (inspired by the RAND/UCLA method), which restricted the process to three rounds, including one large online survey. Furthermore, although the survey evaluated many ISC actives, it did not address different concentrations or potential synergy between combinations of actives. Also, a few actives are not authorized or available in all countries (e.g., benzoyl peroxide, exosomes, growth factors, and hydroquinone).

However, a key strength of this study is the collective clinical experience of 14 experts from 10 countries across 5 continents, combined with an extensive literature review, to provide a broad perspective on appropriate ISC actives for diverse patient populations and procedure categories. All the experts have extensive experience in treating patients with SOC and the use of periprocedural ISC to prevent complications such as PIH.

### 4.2 Conclusions and Future Perspectives

This international consensus provides guidance on selecting ISC actives for various aesthetic procedures at different time points to reduce the risk of irritation, improve wound healing and promote barrier repair to achieve optimal outcomes. ISC selection should be guided by the procedure type, time point of application, and the existing evidence on high‐quality formulations. Actives with the strongest consensus recommendations for ISC across all four procedure categories included ceramides, hyaluronic acid, cholesterol, niacinamide, panthenol, and peptides, while the top‐ranked actives on treatment day and for short‐term aftercare were ceramides, hyaluronic acid, and vitamin C. The consensus group recommended avoiding retinoids and acids on treatment day and for short‐term aftercare during the healing phase due to a risk of irritation and PIH in SOC.

Safety was the primary consideration during the consensus process and the panelists assumed the patients had healthy skin and no specific skin issues. Further research is needed to provide consensus recommendations on ISC actives, concentrations and combinations of actives, tailored to the individual skin type and skin conditions (e.g., photoaging, dyspigmentation, PIH, acne, rosacea, melasma, sensitive skin), as well as the procedure category. Future technological advances (e.g., artificial intelligence tools, imaging, and regenerative techniques) in aesthetic medicine may facilitate personalized ISC strategies to enhance clinical safety and outcomes.

## Author Contributions

Patricia Brieva and Hina Choudhary were involved in the conception and design of the study and data interpretation but were not on the consensus voting panel. All authors contributed to the development of the manuscript, reviewed and approved the final version for publication, and agreed to be accountable for all aspects of the work.

## Ethics Statement

The authors have nothing to report.

## Conflicts of Interest

All panel members were invited by SkinCeuticals. Patricia Brieva and Hina Choudhary are employees of SkinCeuticals.

## Supporting information


**Table S1:** List of Actives Included in the Survey.
**Table S2:** Actives for Which Fewer Than 14 Panelists Responded at One or More Time Points, by Procedure Category.
**Table S3:** Actives Without Consensus by Procedure Category and Time Point.

## Data Availability

The data that support the findings of this study are available from the corresponding author upon reasonable request.
