# Correction to “International Expert Consensus on Integrated Skincare Active Ingredients for Pretreatment and Posttreatment Use With Medical Aesthetic Procedures to Enhance Skin Benefits”

**DOI:** 10.1111/jocd.70989

**Published:** 2026-06-12

Bjerring P , Draelos ZD , Fabi SG , et al. 2026. “International Expert Consensus on Integrated Skincare Active Ingredients for Pretreatment and Posttreatment Use With Medical Aesthetic Procedures to Enhance Skin Benefits.” Journal of Cosmetic Dermatology 25:e70880. 10.1111/jocd.70880.42087526 PMC13145317

In the author list, the middle name of the fifth author was misspelled. The correct author name is “Kwun Cheung Hau.”

The online version of this article has been corrected accordingly.

We apologize for this error.

