# Supplementary material for: International Expert Consensus on Integrated Skincare Active Ingredients for Pretreatment and Posttreatment Use With Medical Aesthetic Procedures to Enhance Skin Benefits
Source: J Cosmet Dermatol. 2026 May 5;25:e70880. doi: 10.1111/jocd.70880 (PMC13145317; doi:10.1111/jocd.70880)
Supplement: Supplementary file 1 — Table S1: List of Actives Included in the Survey. Table S2: Actives for Which Fewer Than 14 Panelists Responded at One or More Time Points, by Procedure Category. Table S3: Actives Without Consensus by Procedure Category and Time Point. [file JOCD-25-e70880-s001.docx]

**Supporting Information**

**Table S1. List of Actives Included in the Survey**

| **Dermocosmetic active ingredient** |
| --- |
| Adenosine |
| Apha-bisabolol |
| Arbutin |
| Azelaic acid |
| Baicalin |
| Bakuchiol |
| Benzoyl peroxide (USA, 2%) |
| Botanical extracts (Thyme/Olive/Cucumber, Eucalyptus leaf) |
| Ceramide |
| Cholesterol |
| Cysteamine |
| Ectoin |
| Exosomes |
| Fatty Acids |
| Ferulic Acid |
| Glutathione |
| Glycolic acid |
| Growth factors |
| Hepes (Hydroxyethylpiperazine Ethane Sulfonic Acid), |
| Hyaluronic acid |
| Hydroquinone (2% USA) or 4% |
| Kojic acid |
| Lactic acid |
| Lactobacillus/Soybean Ferment Extract |
| Laminaria Extract |
| Licorice |
| Niacinamide |
| Panthenol |
| Peptides |
| Polyhydroxy acid (PHA) |
| Phloretin |
| Poly-L lactic acid (PLLA) |
| Proxylane® (C-xyloside) |
| Resveratrol |
| Retinoids |
| Rhamnose |
| Salicylic Acid |
| Silymarin |
| Tranexamic acid |
| Vitamin C (L-ascorbic acid) |
| Vitamin E (Tocopherol) |
| Wild Fruit Flavonoids (Blueberry and Pomegranate extracts) |

**Table S2. Actives for Which Fewer Than 14 Panelists Responded at One or More Time Points, by Procedure Category**

For the active ingredients presented in Figure 1 (main manuscript), the total number of panelist responses at each time point, by procedure category, is shown below for those with fewer than 14 responses at one or more time points. In these cases, consensus was defined as ≥ 75% agreement among respondents.

For each specific procedure category, actives appropriate at all time points are highlighted in green, actives which should be avoided on treatment day are highlighted in pale pink, and actives to be avoided on both treatment day and during short-term aftercare are highlighted in dark pink.

| **ABLATIVE ENERGY-BASED** | | **NON-ABLATIVE ENERGY-BASED** | | **NON-ENERGY-BASED WITHOUT BARRIER DISRUPTION** | | **NON-ENERGY-BASED WITH BARRIER DISRUPTION** | |
| --- | --- | --- | --- | --- | --- | --- | --- |
| **Active** | **Time point, number of responses** | **Active** | **Time point, number of responses** | **Active** | **Time point, number of responses** | **Active** | **Time point, number of responses** |
|  |  | Alpha-bisabolol | Pretreat, 8  Treatment, 9  ST, 9  LT, 8 | Alpha-bisabolol | Pretreat, 8  Treatment, 8  ST, 8  LT, 9 | Alpha-bisabolol | Pretreat, 8  Treatment, 9  ST, 10  LT, 6 |
|  |  | Panthenol | Pretreat, 10  Treatment, 9  ST, 10  LT, 9 | Panthenol | Pretreat, 7  Treatment, 8  ST, 8  LT, 8 | Panthenol | Pretreat, 9  Treatment, 10  ST, 10  LT, 7 |
|  |  | Vitamin E | Pretreat, 9  Treatment, 11  ST, 14  LT, 9 |  |  |  |  |
| Rhamnose | ST, 7 |  |  |  |  |  |  |
|  |  |  |  |  |  | Exosomes (Human/ Animal) | Treatment, 8  ST, 8 |

Abbreviations for time points with missing data: Pretreat = pretreatment, Treatment = Day of treatment, LT = long-term aftercare, ST = short-term aftercare

**Table S3. Actives Without Consensus by Procedure Category and Time Point**

Consensus was defined as follows: appropriate if ≥ 11 panelists (or ≥ 75% of panelists if missing data) voted ‘Often/Always’ or ‘Sometimes appropriate’ (with < 4/14 answers in ‘Never’ or ‘Rarely appropriate’); not appropriate if ≥ 11 panelists (or ≥ 75% of panelists if missing data) voted ‘Never’ or ‘Rarely appropriate’ (with < 4/14 answers in ‘Often/Always’ or ‘Sometimes appropriate’).

All other scenarios (<11 panelists in agreement; < 75% agreement among respondents) were classified as no consensus.

| **ABLATIVE ENERGY-BASED** | | **NON-ABLATIVE ENERGY-BASED** | | **NON-ENERGY-BASED WITHOUT BARRIER DISRUPTION** | | **NON-ENERGY-BASED WITH BARRIER DISRUPTION** | |
| --- | --- | --- | --- | --- | --- | --- | --- |
| **Active** | **Time point** | **Active** | **Time point** | **Active** | **Time point** | **Active** | **Time point** |
| Growth Factors (Human) | Treatment  ST  LT | Adenosine | All time points | Arbutin | ST | Botanical Extracts | Pretreat  Treatment  ST |
| Growth Factors (Plant/Peptide) | ST* | Baicalan | Treatment | Botanical Extracts | All time points | Cysteamine | LT |
| Hydroquinone | ST | Bakuchiol | Pretreat*  Treatment*  ST* | Cysteamine | LT | Exosomes (Plant) | Treatment*  ST* |
| Kojic Acid | ST | Botanical Extracts | Treatment | Exosomes (Human/Animal) | Treatment*  ST* | Ferulic Acid | Treatment  ST |
| Proxylane® (C-xyloside) | Treatment* | Ectoin | Treatment  ST | Exosomes (Plant) | Pretreat  Treatment*  ST* | Growth Factors (Human) | Pretreat*  Treatment*  ST* |
| Retinoids | ST | Fatty Acids | Treatment | Ferulic Acid | Treatment  ST | Growth Factors (Plant/Peptide) | LT* |
| Rhamnose | ST* | Hydroquinone | Pretreat | Growth Factors (Human) | All time points* | Resveratrol | Treatment |
| Silymarin | ST |  |  | Laminaria Extract | Treatment | Vitamin C | Treatment  ST |
| Vitamin C | ST |  |  | Licorice Extract | Treatment  ST | Vitamin E | Treatment |
|  |  |  |  | Resveratrol | Treatment | Wild Fruit Flavonoids | ST |
|  |  |  |  | Vitamin C | Treatment  ST |  |  |
|  |  |  |  | Vitamin E | Treatment |  |  |
|  |  |  |  | Wild Fruit Flavonoids | Treatment  ST |  |  |

*Time points with fewer than 14 panelist responses; no consensus was defined as <75% agreement among respondents.
Abbreviations: Pretreat = pretreatment, Treatment = Day of treatment, LT = long-term aftercare, ST = short-term aftercare
